# Dynamic evolution of osseous structure in osteonecrosis of the femoral head and dynamic collapse risks: a preliminary CT image study

**DOI:** 10.1186/s13018-020-02069-8

**Published:** 2020-11-17

**Authors:** Zeqing Huang, Biao Tan, Hengli Ye, Fanyu Fu, Rongtian Wang, Weiheng Chen

**Affiliations:** 1 The Third Affiliated Hospital of Beijing University of Chinese Medicine, No. 51 Anwai Xiaoguanjie, Chaoyang District, Beijing, 100029 People’s Republic of China; 2 Wangjing Hospital, China Academy of Chinese Medical Sciences, No. 6 Wangjing Zhonghuannan Road, Chaoyang District, Beijing, 100102 People’s Republic of China

**Keywords:** Osteonecrosis of the femoral head, Collapse of the femoral head, Prognosis estimation, Medical imaging analysis, Computer tomography

## Abstract

**Background:**

Collapse risk of osteonecrosis of the femoral head (ONFH) is estimated mainly based on static indicators, including lesion size and lesion location, but bone repairing is a dynamic process that lasts for years. The present study attempted to analyze the dynamic evolution of the osseous structure and its correlation with radiographic progression.

**Methods:**

This retrospective study included 50 hips with ONFH from 50 patients. Participants were divided into the non-collapse group (*n* = 25) and the collapse group (*n* = 25). Original files of the initial computed tomography (CT) images were imported into imaging processing software for morphology analysis. The volume of sclerotic bone, the volume of soft tissue, and bone mineral density (BMD) were calculated. The linear correlations between the aforementioned indicators and the disease duration were estimated. The logistic regression analysis was conducted to evaluate the correlation of these indicators with the radiographic progression. Receiver operating characteristic (ROC) analysis was used to evaluate these indicators’ prediction performance.

**Results:**

The volume of sclerotic bone and the BMD grew with disease duration, but the volume of soft tissue decrease. The logistic regression analysis found that the volume of sclerotic bone and the BMD were statistically associated with radiographic progression. The ROC analysis found that the regression model, which integrated the volume of sclerotic bone and the BMD, had satisfactory performance in predicting radiographic progression.

**Conclusion:**

The present study suggested a dynamic evolution of the osseous structure and a dynamic variation trend of the collapse risk in ONFH. The volume of sclerotic bone and the BMD might serve as further prognostic indicators when estimating the collapse risk.

## Introduction

Osteonecrosis of the femoral head (ONFH) is a disabling condition due to the femoral head’s compromised blood supply [1]. ONFH is one of the main reasons to cause rapid destruction of the hip joint [2]. Patients with ONFH will finally lose their walking function starting from feeling progressive symptoms, including groin pain and restricted physical function [3]. Since ONFH primarily affects the young- and middle-aged generation, some patients who secondarily develop severe hip osteoarthritis have to undergo total hip arthroplasty (THA) at their young ages [4, 5]. Overall, THA is not an optimal solution due to the limited implant lifespan and potential revision surgery complications [6, 7].

Appropriate treatments before the onset of femoral head collapse might hopefully help delay or cease the progression to collapse of the femoral head, and thus might finally delay or avoid THA [8]. The collapse risk of the femoral head is the main criterion for treatment decision-making. Currently, no optimal treatments exist, but operative treatments, specifically named as joint-preserving procedures, are strongly recommended for patients with increased collapse risk [9]. Lesion size and lesion location are now accepted as critical prognostic indicators [10–12]. Necrotic lesions with a larger size, or occupying the lateral part of the femoral head, are more likely to progress to collapse.

On the other hand, great efforts have focused on the basic research of the osseous structure in ONFH. It is believed that the change in the osseous structure is closely and directly associated with the progression to collapse [13, 14]. In clinical practice, the characteristics of osseous structures are seldom considered when evaluating the collapse risks [15]. Questions thus arise when comparing the collapse risks of different patients with familiar lesion size and lesion location, as well as the same patient in different follow-up time points. If only considering the lesion size and lesion location, the collapse risks in these conditions should be the same. Obviously, the osseous structures inside the femoral head vary. Opposite opinions have been put forward regarding the effect of osseous structure on the progression of collapse [16–18]. One of the reasons for the controversial debate, in our opinion, is that collapse risk would be static when only considering the lesion size and lesion location, since the lesion size and lesion location are stable features of the necrotic lesion. On the contrary, we hypothesize the collapse risk is dynamic, since the repairing process of osseous structure lasts for years [1].

Although the boundary of the necrotic lesion is popularly discussed in previous studies, there are not totally objective methods to segment the lesion yet. Late researches reported that even using imaging processing software, the segmentation process of the necrotic lesion on magnetic resonance imaging (MRI) still needs human assistance [12, 19]. In the present study, we abandon the traditional method to analyze the disease by segmenting the necrotic lesion. We analyze the morphology and evolution of the osseous structure within the entire femoral head in ONFH patients, as well as their correlation with the radiographic progression.

## Participants and methods

This retrospective cohort study included 50 hips with ONFH from 50 patients. We recruited five other healthy participants as normal controls for the comparison with the patients with ONFH hips in the morphology analysis process. All participants consented to participate in this study. According to the Association Research Circulation Osseous (ARCO) classification system [20], patient-participants have confirmed diagnosis of ONFH based on the distinctive radiographic features seen on MRI, including a focal serpentine low signal line with fatty center on T1-weighted image (reactive interface line) and serpiginous peripheral dark line and inner bright line on T2-weighted image (double line sign). The inclusion criteria were ARCO stage II or ARCO stage IIIa lesions, a modified Keboul angle over 250°, and did not receive operative treatment. The other inclusion criteria for this retrospective study were that patients should have undergone computed tomography (CT) scans for at least twice. The interval between the two series of CT images selected for the following analysis should be 1 year. The first series of CT images were to confirm the inclusion criteria and also used for software analysis. The second series of CT images were used to confirm the radiographic progression. The exclusion criteria were that patients were still undergoing corticosteroid therapy or could not quit drinking alcohol.

The present study included 25 hips (non-collapse group) without radiographic progression and 25 hips (collapse group) with radiographic progression at the end of a 1-year follow-up. Radiographic progression referred to the progression of collapse, including the new occurrence of collapse (from ARCO stage II to ARCO stage III) and the new deterioration of collapse (from ARCO stage IIIa to ARCO stage IIIb or IIIc). The femoral head collapse referred to the subchondral fracture or depression of the femoral head seen on CT images.

Among these 50 patients, there were 28 males and 22 females with an average age of 38.12 ± 10.14 years (range, 18 to 60 years, see Table 1). The reasons for ONFH were alcohol abuse in 18 cases, corticosteroid use in 21 cases, and idiopathic in the remaining 11 cases. Based on the determination criteria of the modified Kerboul’s angle [12, 21], the mean Kerboul’s angle was 285.24 ± 36.40° (range, 250.94 to 444.54°). According to the ARCO classification, 29 hips were classified as stage II (abnormal plain radiographs without collapse or a crescent sign) and 21 hips as stage IIIa (abnormal plain radiographs with depression in the femoral head less than 2 mm or with a crescent sign). To analyze the dynamic evolution of the osseous structure, we defined the disease duration as the time from the onset of hip symptoms up to the time patient received the initial CT scanning in the present study. The mean disease duration was 17.52 ± 10.84 months (range, 3 to 42 months).

**Table 1:** Basic characteristic of the patient participants

Characteristic	Amount (%) of patients, *n* = 50
Age, years, mean (SD)	38.12 (10.14)
Gender	
Male	28 (56)
Female	22 (44)
Etiology	
Alcohol	18 (36)
Corticosteroid	21 (42)
Idiopathic	11 (22)
Kerboul’s angle, degree, mean (SD)	285.24 (36.40)
ARCO classification	
II	29 (58)
IIIa	21 (42)
Disease duration, months, mean (SD)	17.52 (10.84)

*SD* standard deviation

*Unless stated otherwise

### CT images analysis

The Mimics Medical 21.0, a medical imaging processing software, was applied in the present study for virtual anatomic study. The masks of the proximal femur were created according to the threshold value of the Hounsfield unit (HU) of adult bone. HU is a standardized unit used in CT images to express the CT number of different tissues [22]. Then the three-dimensional (3D) model of the proximal femur was calculated. We conducted virtual osteotomy at the basilar part of the femoral head to segment the femoral head from the femoral neck. The polylines of the 3D model of the femoral head were calculated, and the masks of the femoral head were created based on these polylines.

In the present study, the osseous structure of the femoral head was classified as three kinds of tissue, including the sclerotic bone, the cancellous bone, and the soft tissue. First, we created the masks of the aforementioned three kinds of tissues on the original CT images according to their different HU ranges. Boolean operation was conducted to segment the three different kinds of tissues within the femoral head, by calculating the intersection part of the mask of the femoral head and the masks of the three kinds of tissues on the original CT images. The 3D model, as well as the volume of the different tissues, was calculated respectively.

The 3D models of the femoral head were imported into the 3-Matic Medical 13.0, a 3D model processing software, to create uniform and volume mesh. Then, the mesh models of the femoral head were imported back into the Mimics software, and the calculation of bone mineral density (BMD) was conducted according to the experience formula method reported in previous studies [23, 24].

### Statistical analysis

We estimated the linear correlation between the disease duration and the volume of sclerotic bone, the volume of soft tissue, and the BMD. We did not analyze the volume of the cancellous bone, since the cancellous bone is the main and normal tissue of the femoral head. Logistic regression analysis was used to evaluate the relationship between radiographic progression and the volume of sclerotic bone, the volume of soft tissue, and the BMD. The receiver operating characteristic curve (ROC curve) analysis was conducted to estimate the performance of the studied indicators on the prediction of radiographic progression.

## Result

### Morphology analysis

**Fig. 1:**
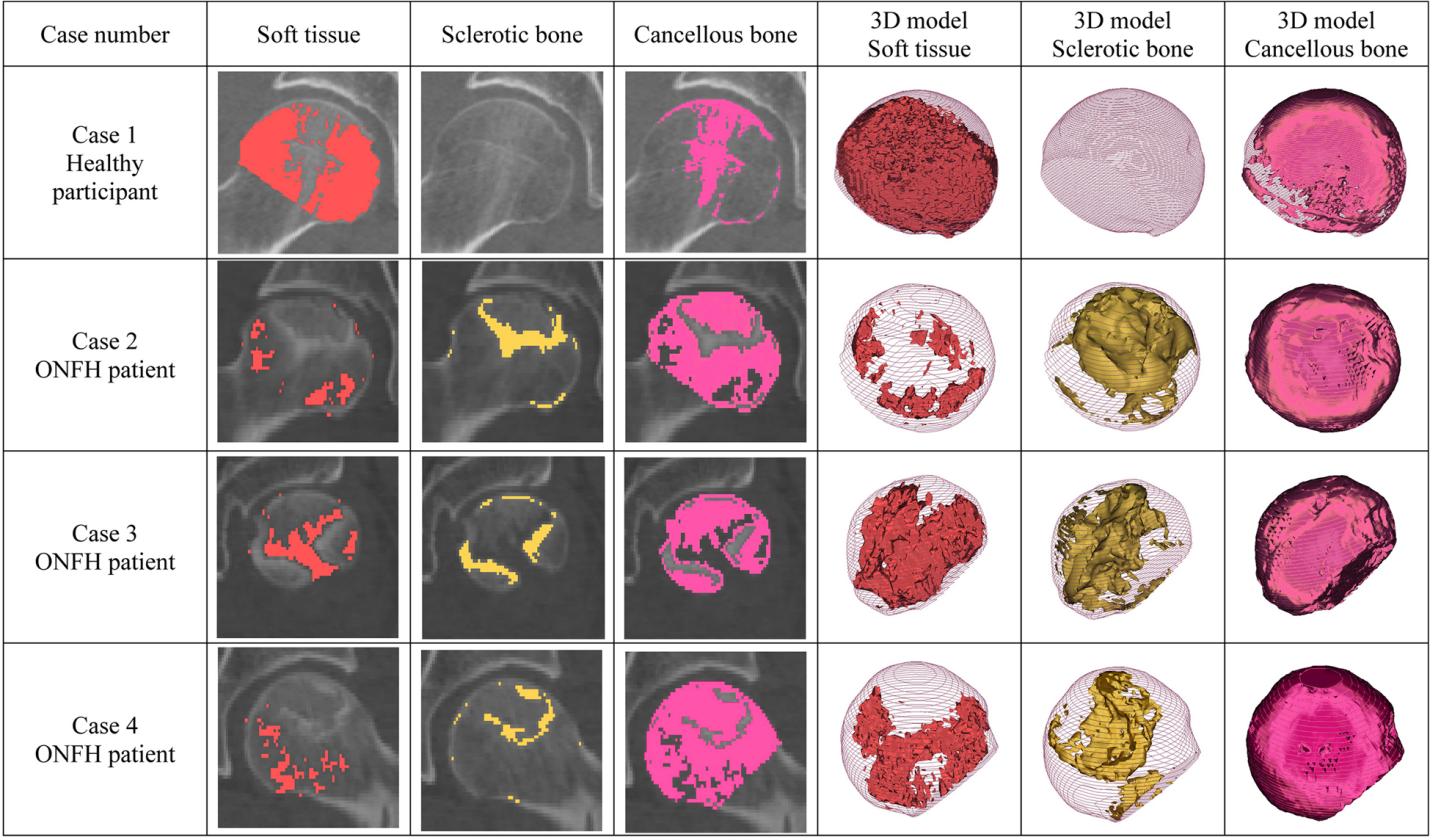
Morphology analysis of the femoral head

The osseous structure of the hips with ONFH was quite different from the healthy hips (see Fig. 1). The development of sclerotic bone within the necrotic lesion was a significant difference from the healthy controls. However, according to the present study, the necrotic lesions were a mixture of sclerotic bone and cancellous bone, and sometimes soft tissue, instead of only sclerotic bone. The necrotic lesions tend to develop a sclerotic boundary, but we observed that the sclerotic boundary was always discontinuous. On the other hand, the distribution of soft tissue in ONFH hips also seems abnormal compared to the healthy hips. The femoral head with ONFH was observed to contain less soft tissue. Also, the decrease of soft tissue was found outside the lesion. Hence, ONFH affects the osseous structure of the whole femoral head instead of only the necrotic lesion.

### Osseous structure time variation

**Fig. 2:**
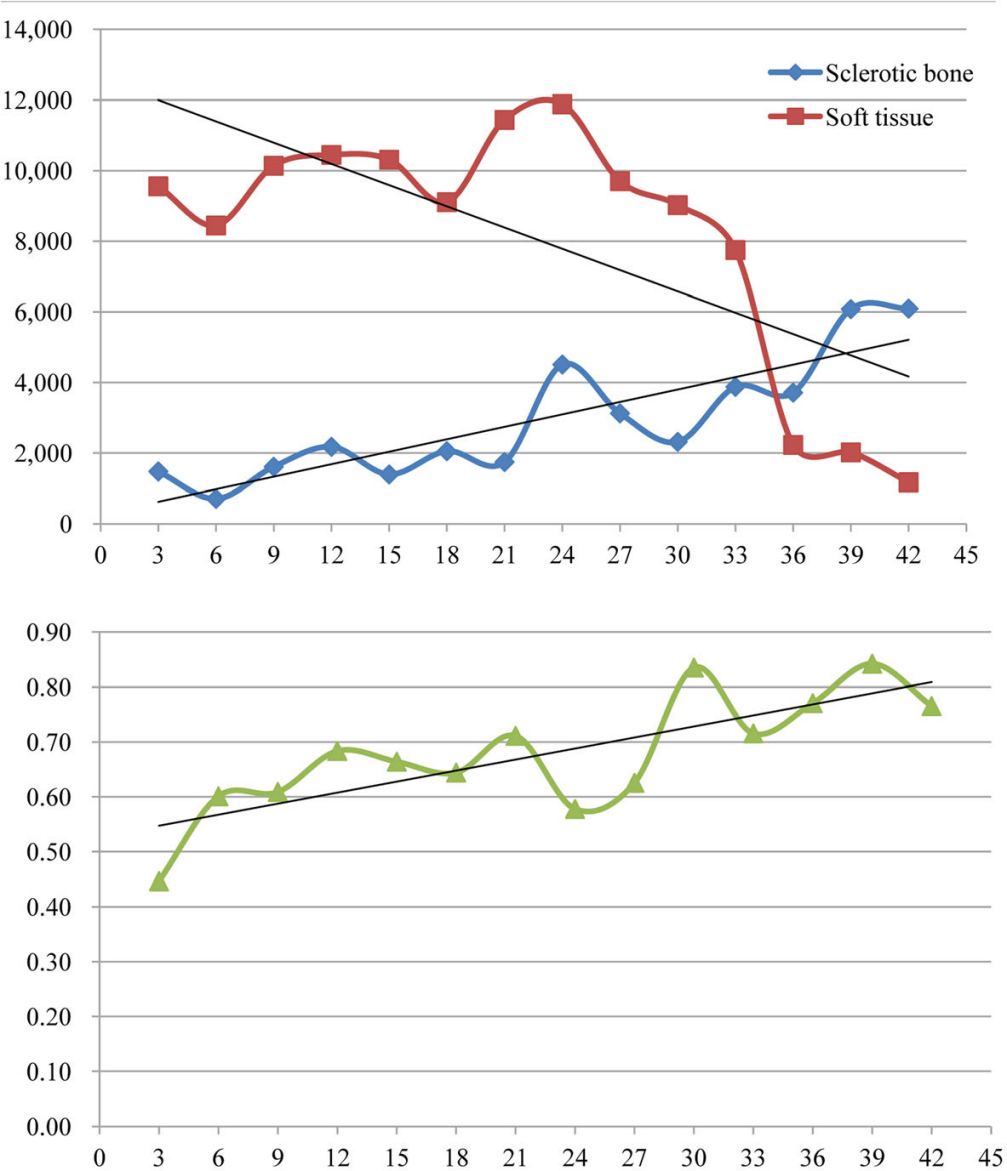
Line charts showing the dynamic evolution of osseous structure indicators with disease duration. Each dot in the chart represents the mean value of osseous structure indicators at a certain time point of the disease duration

Significant statistical differences were observed when estimating the linear correlation between the disease duration and the volume of sclerotic bone, the volume of soft tissue, and the BMD. The volume of sclerotic bone and the BMD tend to increase with disease duration, but the volume of soft tissue tends to decrease (see Fig. 2 and Table 2). The *R*^2^ value represents the proportion of each osseous structure indicator explained by the disease duration. However, each single osseous structure indicator did not seem to be influenced only by disease duration, since we observed that both *R*^2^ values and adjusted *R*^2^ values were limited.

**Table 2:** Linear correlation between disease duration and osseous structure indicators

Indicator	*B*	*F*	*P*	*R*^2^	Adjusted *R*^2^
Volume of sclerotic bone (mm^3^)	126.424	37.905	< 0.001	0.441	0.430
Volume of soft tissue (mm^3^)	− 144.674	4.648	0.036	0.088	0.069
Bone mineral density (g/L)	0.005	11.929	0.001	0.199	0.182

### Clinical significance of osseous structures

Binary logistic regression analysis (see Table 3) found the volume of sclerotic bone and the BMD were significantly correlated with the radiographic progression, and the volume of soft tissue was removed from the regression model due to the lack of statistical correlation. An increase in the BMD indicated a decrease in the risk of progressive collapse, but we only observed a neutral effect of the volume of sclerotic bone. Therefore, the regression model that integrated the BMD and the volume of sclerotic bone was used as a composite index to predict radiographic progression in further analysis. The equation of the logistic regression analysis was as follows:

$$ \mathrm{Logit}(P)=5.137+0.001\times \mathrm{volume}\ \mathrm{of}\ \mathrm{sclerotic}\ \mathrm{bone}-9.674\times \mathrm{bone}\ \mathrm{mineral}\ \mathrm{density} $$

**Table 3:** Logistic regression analysis of radiographic progression and osseous structure indicators

Indicator	*P*	OR	95% CI
Volume of sclerotic bone	0.020	1.001	1.000~1.001
Bone mineral density	0.008	< 0.001	0.000~0.083
Constant	0.019	170.256	\

And the probability equation using the regression model was as follows:

$$ P=\frac{e^{\log \mathrm{it}(P)}}{1+{e}^{\log \mathrm{it}(P)}} $$

### Prediction performance for radiographic progression

**Fig. 3:**
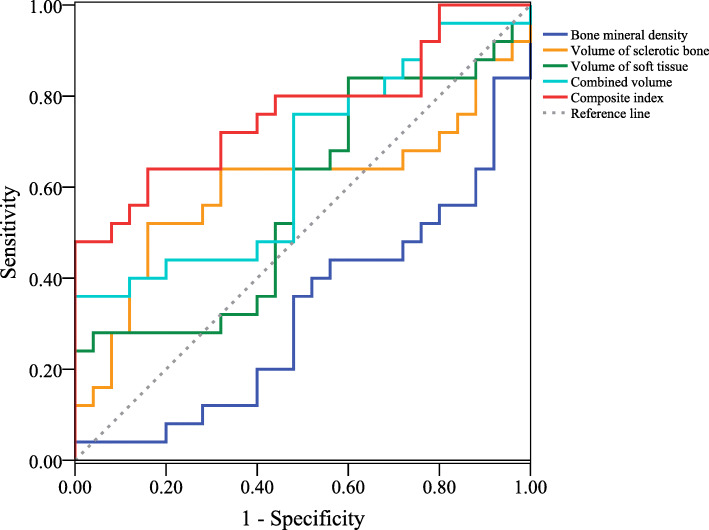
Line chart showing the receiver operating characteristic curves of different collapse prediction methods

The area under the curves showed in Fig. 3 represented the capacity of different prediction methods with osseous structure indicators on predicting the radiographic progression. Although the area under the curve (AUC) was over 50.00% in prediction models including the volume of sclerotic bone, the volume of soft tissue (see Table 4), and the combined volume of sclerotic bone and soft tissue, there was no statistical difference. Statistical difference was observed when predicting radiographic progression with only the BMD, but the prediction performance was limited. We found the regression model integrating the BMD and the volume of sclerotic bone, as a composite index, had a satisfactory performance on predicting radiographic progression, with an AUC of 76.50%.

**Table 4:** Area under the ROC curve of prediction methods with different osseous structure indicators

Indicator	Area under ROC curve	*P*	95% CI
Bone mineral density	0.323	0.032	0.174~0.473
Volume of sclerotic bone	0.600	0.225	0.434~0.766
Volume of soft tissue	0.573	0.377	0.410~0.736
Combined volume (sclerotic bone and soft tissue)	0.654	0.061	0.501~0.808
Composite index (logistic regression model integrating bone mineral density and volume of sclerotic bone)	0.765	0.001	0.630~0.900

## Discussion

The present study reported a novel approach to analyze the evolution of osseous structure within the femoral head affected by ONFH. We found ONFH affected not only the necrotic lesion but also the osseous structure outside the lesion. Additionally, the so-called lesion boundary (sclerotic boundary) was not always continuous, which was the main reason that the machine segmentation of the necrotic lesion still needs human assistance. The present study tried to conquer the technological limitation by analyzing the osseous structure without segmenting the necrotic lesion. We found that the volume of sclerotic bone, the volume of soft tissue, and the BMD of the entire femoral head changed with disease duration. The volume of sclerotic bone, when integrated with the BMD, might provide expectable performance on predicting radiographic progression.

Since ONFH primarily affects a limited subchondral part of the femoral head, instead of the entire femoral head, for a long time, researchers had been actively discussing the criteria to segment and classify the necrotic lesion [12]. To date, clinical practitioners get used to categorizing the necrotic lesions into large lesions, medium lesions, and small lesions [25, 26]. In real-world clinical practice, plain radiographs are the most used imaging modality to estimate the lesion size. At the same time, it is also generally acknowledged that plain radiographs are not an accurate modality to measure the lesion size [12]. A low signal line with a fatty center on T1-weighted MRI is a specified feature to confirm the diagnosis of ONFH, and a low signal line is frequently used as the criterion to segment the necrotic lesion. However, the low signal line is not always clear and continuous. The automatic process of segmenting the necrotic lesion, in fact, still relies on human assistance [12, 19]. CT is a better modality to observe the evolution of the osseous structure, and the sclerotic boundary is used as a segmentation criterion of the lesion. According to our study, the sclerotic boundary changed over time; meanwhile, it shared features similar to the low signal line that it was also not always clear and continuous.

Therefore, the present study analyzes the evolution of osseous structures within the femoral head instead of segmenting and analyzing the necrotic lesion. CT images are sensitive to identify the change of osseous structures that are technologically reflected by the HU [27]. HU is used as the tissue classification criteria to segment different tissues and organs based on their thresholds. In the present study, the tissues within the femoral head are classified into sclerotic bone, cancellous bone, and soft tissue, with HU thresholds different from each other. After the automatic identification of HU in the Mimics software, the three different tissues can also be automatically segmented based on their distinct HU thresholds.

The present study reports osseous structure-time variations that the volume of sclerotic bone and the BMD grow with disease duration, and the volume of soft tissue decreases. Most classification systems of ONFH acknowledged that the osseous structure within the lesion changes with time, like in ARCO stage I, the osseous change is absent on plain radiographs, but in ARCO stage II, sclerosis or cyst can be seen. However, the current classification systems do not take into consideration the dynamic evolution of the osseous structure. Previous research supported that the change of the osseous structure is the root cause of the progression of femoral head collapse [13, 18]. Indeed, lesion size and lesion location measurements are based on the significant change of the osseous structure. There are arguments on the effect of changing in the osseous structure. Yu et al. reported that the sclerotic boundary might prevent the collapse of the femoral head [17], but Utsunomiya et al. concluded that the onset of the sclerotic boundary might trigger the progression to collapse [18]. In our opinion, these valuable studies are generally isolated and static studies, since they ignore the dynamic evolution process of the osseous structure.

The present study found that the volume of sclerotic bone and the BMD were closely associated with the radiographic progression, which has potentials for the prediction of radiographic progression when being integrated together. Moreover, as mentioned above, the volume of sclerotic bone and the BMD grow with disease duration. This may explain why different conclusions were made when discussing the effect of sclerotic boundary on the radiographic progression. Previous researchers analyzed the sclerotic rim in a single follow-up time point, but the sclerotic rim would grow with time.

There are several limitations to the study. According to our analysis, disease duration could only explain the changes in the osseous structure to a limited extent (Table 2). The bone repair process in ONFH remains largely unknown. Several different biological processes are involved in this process, including oxidative stress, angiogenesis, bone turnover, and inflammation [2]. Our finding was consistent with the present understanding of the bone repair process. Secondly, only a small sample size of participants without a history of operative treatment was included in the present study, since most patients with large lesions should have undergone operative treatments. Thirdly, we abandoned to analyze the evolution of the same lesions of the same participants, since it is not ethical to perform CT scannings on the same participant in the interval of every 3 months. Fourthly, the radiographic progression is defined by the collapse or progressive collapse in the follow-up duration of 1 year. Thus, the prediction performance of the integrated indicators should be strictly limited to a short term of not more than 1 year. Lastly, this was a retrospective study, and we only included patients without collapse or with mild collapse. Hence, the evolution pattern of the osseous structure reported in the present study should be carefully reconsidered before it was used for the evaluation of patients with severe collapse.

In conclusion, the present study used a classification segmentation method to analyze the osseous structure within the femoral head instead of segmenting and analyzing the necrotic lesion. We found the dynamic evolution process of the osseous structures that the volume of sclerotic bone and the BMD grow with disease duration, but the volume of soft tissue decreases. The volume of sclerotic bone and the BMD are closely associated with the occurrence and progression of collapse. Hence, the collapse risk might change with disease duration due to the dynamic evolution of the osseous structure. Our findings might complement the static collapse risk evaluation method which only includes lesion size and lesion location, and help explain the controversial debates regarding the effect of osseous structure on radiographic progression.

## Data Availability

Raw data were generated at Wangjing Hospital. Derived data supporting the findings of this study are available from the corresponding author on request.

## Abbreviations

ONFH: Osteonecrosis of femoral head
CT: Computed tomography
BMD: Bone mineral density
ROC analysis: Receiver operating characteristic analysis
THA: Total hip arthroplasty
MRI: Magnetic resonance imaging
ARCO: Association Research Circulation Osseous
HU: Hounsfield unit
3D: Three-dimensional
AUC: Area under the curve
